# Carbon Emission Evaluation of CO_2_ Curing in Vibro-Compacted Precast Concrete Made with Recycled Aggregates

**DOI:** 10.3390/ma16062436

**Published:** 2023-03-18

**Authors:** David Suescum-Morales, Enrique Fernández-Ledesma, Ágata González-Caro, Antonio Manuel Merino-Lechuga, José María Fernández-Rodríguez, José Ramón Jiménez

**Affiliations:** 1 Área de Ingeniería de la Construcción, Escuela Politécnica Superior de Belmez, Universidad de Córdoba, 14240 Córdoba, Spain; p02sumod@uco.es (D.S.-M.); efledesma@uco.es (E.F.-L.); ammlechuga@uco.es (A.M.M.-L.); 2 Área de Química Inorgánica, Escuela Politécnica Superior de Belmez, Universidad de Córdoba, 14240 Córdoba, Spain; q32gocaa@uco.es

**Keywords:** carbon emission evaluation, CO_2_ curing, waste recycling, CO_2_ sequestration, construction and demolition waste

## Abstract

The objective of the present study was to explore three types of vibro-compacted precast concrete mixtures replacing fine and coarse gravel with a recycled/mixed concrete aggregate (RCA or MCA). The portlandite phase found in RCA and MCA by XRD is a “potential” CO_2_ sink. CO_2_ curing improved the compressive strength in all the mixtures studied. One tonne of the mixtures studied could be decarbonised after only 7 days of curing 13,604, 36,077 and 24,635 m^3^ of air using natural aggregates, RCA or MCA, respectively. The compressive strength obtained, XRD, TGA/DTA and carbon emission evaluation showed that curing longer than 7 days in CO_2_ was pointless. The total CO_2_ emissions by a mixture using CO_2_ curing at 7 days were 221.26, 204.38 and 210.05 kg CO_2_ eq/m^3^ air using natural aggregates, RCA or MCA, respectively. The findings of this study provide a valuable contribution to carbon emission evaluation of CO_2_ curing in vibro-compacted precast concrete with recycled/mixed concrete aggregates (RCA or MCA). The technology proposed in this research facilitates carbon capture and use and guarantees enhanced compressive strength of the concrete samples.

## 1. Introduction

Ordinary Portland cement (OPC) is the most widely used material in construction worldwide, with a global consumption between 1930 and 2013 of 76.2 billion tonnes [1,2]. The manufacture of OPC produces a large amount of CO_2_ (1 tonne of cement emits approximately 1 tonne of CO_2_), which means that 8% of the world’s total CO_2_ emissions are related to the cement industry [3,4,5]. Scientists must therefore work together to reduce/reuse the CO_2_ emissions produced by OPC manufacturing.

Global warming and climate change are growing problems. According to the Intergovernmental Panel on Climate Change (IPCC) [6], the increase in the earth’s surface temperature will lead to dire consequences, and CO_2_ emissions are the main cause of global warming [7,8]. Carbon capture storage (CCS) and carbon capture utilisation technologies (CCU) are among the many ways to reduce CO_2_ emissions [9]. The cost of emitting CO_2_ in the European Union is approximately 80 €/t CO_2_, although this will increase due to current policies [10]. With the implementation of CCU, instead of costing money, CO_2_ would become a source of income.

It has been demonstrated recently that CO_2_ curing of cement-based materials converts gaseous CO_2_ into solid calcium carbonate [11,12,13], also called mineral carbonation technology (MCT), and is considered to be a cost-effective and environmentally-friendly method of capturing and storing CO_2_ [14]. CO_2_ sequestration by mineral carbonation and its subsequent conversion into a product for the construction industry is one of the most representative examples of MCT [15]. Several studies have applied the CO_2_ curing concept to produce commercial building material: Several authors [16,17,18] carbonated different types of slag into building material; Zhen et al. [19] directly used the CO_2_ generated by a cement factory (flue gas carbonation) to cure cement products; Suescum-Morales et al. [5,12,20] studied the application of carbonation in different forms of mortars intended for use as unreinforced construction products up to 7 days of curing. There has been no application of carbonation for longer curing ages, for example, up to 28 days, and studies along these lines would fill many gaps in our current knowledge. Carbonation mainly involves a reaction between CO_2_ and calcium silicate phases [21]. Equations (1) and (2) are usually related to accelerated carbonation at early ages. Equations (3)–(5) are normally related to the durable carbonation of concrete, although they also occur when there is forced carbonation [22,23]. The main equations are presented in Equations (1)–(5):

(1) $$3\left(3CaO\cdot {SiO}_{2}\right)+\left(3-x\right){CO}_{2}+yH_{2}O\to xCaO\cdot {SiO}_{2}\cdot yH_{2}O+\left(3-x\right){CaCO}_{3}$$

(2) $$2\left(2CaO\cdot {SiO}_{2}\right)+\left(2-x\right){CO}_{2}+yH_{2}O\to xCaO\cdot {SiO}_{2}\cdot yH_{2}O+\left(2-x\right){CaCO}_{3}$$

(3) $${Ca\left(OH\right)}_{2}+{CO}_{2}\to {CaCO}_{3}+H_{2}O$$

(4) $$C_{x}{SH}_{y}+z{CO}_{2}\to C_{\left(x-z\right)}{SH}_{y}+z{CaCO}_{3}$$

(5) $$C-S-H+{CO}_{2}\to {CaCO}_{3}+{SiO}_{2}+H_{2}O$$

The rapid development and constant growth of the construction sector generate a large number of recycled aggregates from construction and demolition waste (RAC&D) [24,25], which is around 30 billion tonnes per year. The reuse of masonry waste, such as recycled aggregate, was a highly relevant topic to the scientific community from the early 1970s [26,27]. The main reasons for this interest include the avoidance of depositing such waste and the overexploitation of natural aggregates (NA) [28,29]. Within RAC&D, there are different types of aggregates, depending on their origin (in very short form) [12,20]: recycled ceramic aggregates (ceramic waste); recycled concrete aggregates (concrete waste) and mixed recycled aggregate (a mixture of the two above. RAC&D is usually of poorer quality than NA, which results in poorer properties of the resulting mixtures. This is due to RAC&D having adhered to the old mortar with lower density, higher porosity, lower crush resistance, higher water absorption value and weaker interfacial transition zones (ITZs) [5,12,30,31]. Therefore, the development of new techniques to improve the quality of RAC&D is on the rise [27,32,33,34,35,36,37], among which are those that improve the quality of RAC&D by using CO_2_ (accelerated carbonation), which seems to be a very promising technology [38,39,40,41].

To apply CO_2_ curing at mixtures using RAC&D at a commercial scale, the environmental impact of accelerated carbonation treatment must be considered, since the energy consumption required is usually significant [41,42]. A life cycle assessment (LCA) can be carried out to quantify this impact [43,44,45], or a somewhat simpler form of CO_2_ footprint assessment can be carried out [46,47,48]. There have been several studies conducting the comparative LCA analysis between NA and RAC&D [49], and, although the ISO 14040 standard regulates the definition and selection criteria of functional units, the units chosen for investigating concrete products are insufficient to reflect concrete in terms of its environmental impact [50]. Most of the studies found only calculate the CO_2_ sequestration capacity of CO_2_ cured samples, without taking into account the carbon footprint of the curing process itself [5,12,25,51]. However, CO_2_ sequestration of cement-based waste materials is a multi-process activity with consumption of energy: demolition sector, recycling sector and carbon capture sector, among others [52]. There are no studies that calculate CO_2_ sequestration in mixtures made with RAC&D through thermogravimetric analysis, nor are there studies that perform a carbon footprint assessment considering both the materials used and the curing conditions and time. Studies along these lines can also fill this knowledge gap.

The objective of the present study was to explore three types of vibro-compacted precast concrete mixtures replacing fine and coarse gravel with two types of RAC&D. CO_2_ curing for 1, 3, 7, 14 and 28 days was employed to improve the compressive strength and CO_2_ sequestration. X-ray diffraction (XRD) of the hardened samples was measured to analyse the effect of CO_2_ curing for the duration of 28 days of curing. The carbon emission evaluation was performed using thermogravimetric analysis for the CO_2_ sequestration and, and a CO_2_ footprint assessment for the different mixtures and time/curing conditions.

## 2. Materials and Methods

### 2.1. Raw Materials

**Figure 1:**
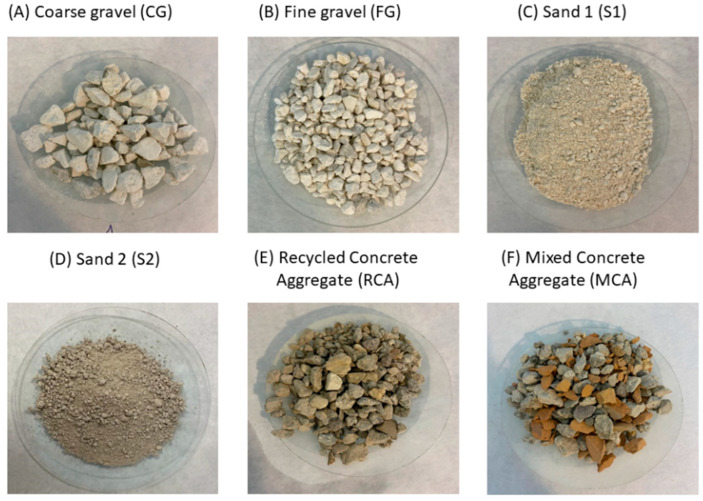
Images of the aggregates used (**A**) CG, (**B**) FG, (**C**) S1, (**D**) S2, (**E**) RCA and (**F**) MCA.

**Figure 2:**
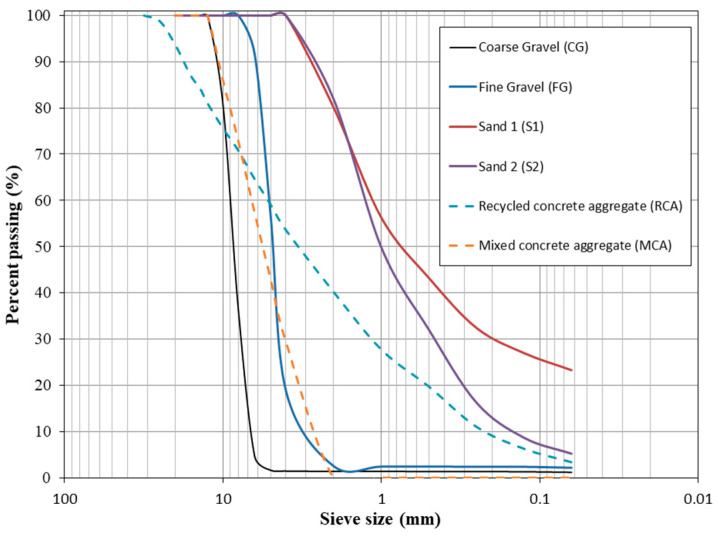
Original particle size distribution of aggregates used.

In this study, two types of natural coarse aggregates and natural fine aggregates were used: coarse gravel (CG), fine gravel (FG), sand-1 (S1) and sand-2 (S2). Two types of recycled aggregate were also used: recycled concrete aggregate (RCA) and mixed concrete aggregate (MCA). Figure 1 shows the aspect of the aggregates used. These aggregates were taken from a quarry in Cordoba (Spain). The difference between RCA and MCA was that MCA included pieces of ceramic bricks. Figure 2 shows the original particle size distribution of all the aggregates used in this study [53]. The skeletal density and water absorption were measured according to UNE-EN-1097-6:2013 [54]. Table 1 shows the basic physical parameter indicators of the aggregates and a CEM II/A-V 42.5 R was used [55]. The mixing water was tap water with polycarboxylate-based product from BASF (Glenium 3030 NS) as a plasticizer (1210 kg/m^3^).

### 2.2. Aggregate Preparation and Mix Design

**Figure 3:**
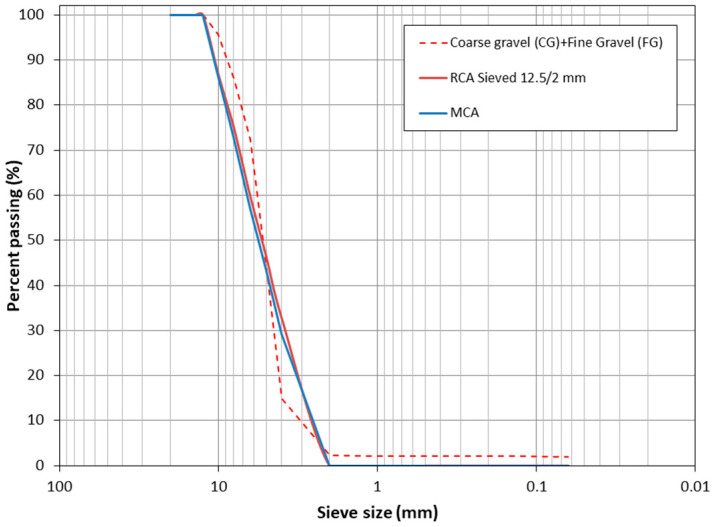
Particle size distribution of CG + FG, RCA sieving and MCA.

The aim was to replace coarse aggregates (CG and FG) with RCA and MCA. However, according to Figure 2, the particle sizes appear to be very different. Therefore, after an experimental and iterative process, it was decided to make a mixture of 22.22% CG and 77.78% FG (with respect to the sum of CG and FG). RCA had very large particle sizes (larger than 12.5 mm) and a very large proportion of finer particles (smaller than 2 mm). Therefore, in order to make the size of MCA and RCA similar, sieving was carried out. RCA material was sieved by 12.5 and 2 mm, rejecting the upper and lower parts, respectively. For MCA, no sieving was required. With this combination, the particle sizes were very similar for the composition of GC + FG, RCA and MCA, as shown in Figure 3.

The proportions of concrete mixtures are given in Table 2. The aim of the mixtures was that they should be demoulded immediately. Therefore, a low w/c ratio was used (w/c = 0.4). For the reference mixture (named CONTROL), the water saturation was calculated according to the values of water absorption shown in Table 1. It can be observed that the composition of CG and FG represented 22.22 and 77.78%, respectively (with respect to the sum of CG and FG).

For the mixture substituting 100% GC + FG by RCA (named M-100-RCA), the amount was calculated as shown in Equation (6). The volumes of both CG and FG (volumetric substitution) were taken into account. The same procedure was followed for the mixture substituting 100% GC + FG by MCA (named M-100-MCA), as shown in Equation (7). To calculate these quantities and the water absorption, the data shown in Table 1 was again used.

(6) $$Amount\;of\;RCA=\frac{\left(\gamma_{RCA}\cdot {Amount}_{CG}\right)}{\left(\gamma_{CG}\right)}+\frac{\left(\gamma_{RCA}\cdot {Amount}_{FG}\right)}{\left(\gamma_{FG}\right)}$$

(7) $$Amount\;of\;MCA=\frac{\left(\gamma_{MCA}\cdot {Amount}_{CG}\right)}{\left(\gamma_{CG}\right)}+\frac{\left(\gamma_{MCA}\cdot {Amount}_{FG}\right)}{\left(\gamma_{FG}\right)}$$

### 2.3. Concrete Mixing Procedure and Casting

The procedure for the mixing was as follows: the coarse aggregates (CG and FG or RCA or MAC) were added and mixed for 1 min, and then the fine aggregates (S1 and S2) were added and mixed for another minute. The saturation water and all aggregates were mixed for 10 min, the effective water together with the plasticizer was added and then the cement was added. A mixing time of 5 min was carried out. The mixer used was a professional electric mixer (Inhersa X155, Inhersa company, Castellón, Spain). For all the mixes, the results of the slump test were 0 mm [56], which indicated a dry consistency or S1 class, according to Eurocode 2 [57,58]. Cube samples of 100 mm were cast.

**Figure 4:**
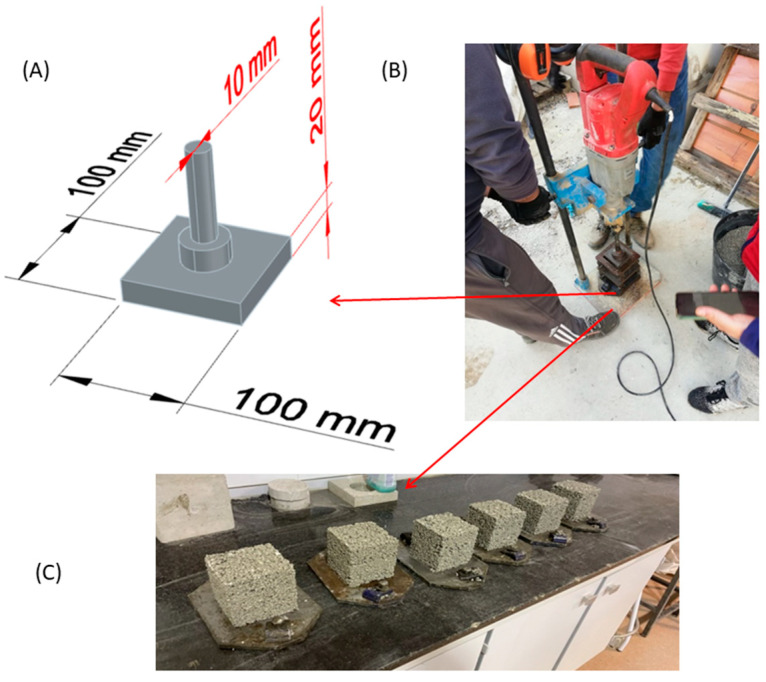
(**A**) 3-D modelling of the special part used to compact the manufactured concrete, made by the authors; (**B**) Hammer used and (**C**) Specimens demoulded immediately after being manufactured.

Due to the very dry consistency of the concretes obtained, vibro-compaction with a Kango vibration hammer (Milwaukee Kango 900 S) was applied. The 100 × 100 mm cubic moulds were filled in 2 batches, with a vibro-compaction of 10 s in each of these batches. The hammer used as well as the 3-D modelling of the specially fabricated steel part for this procedure by the authors is shown in Figure 4. The aim was to follow a procedure similar to that used in an unreinforced precast plant [59].

### 2.4. Curing Conditions and Test Methods

Once the samples were demoulded, they were subjected to:

- Conventional climatic chamber (CCC): 20 °C and 65% relative humidity. The CO_2_ level under this environment was equivalent to atmospheric conditions (≈0.04%);
- CO_2_ climatic chamber (CO_2_CC): For this environment a Climacell 707-Evo (MMM Group, Planegg, München, Germany) with a CO_2_ level of 5% (99.995% purity, supplied by Linde) and 20 °C with 65% relative humidity. The pressure was ambient.

According to the manufacturer, the maximum power of both pieces of equipment was 300 W, but with these conditions, a consumption of about 0.15 kW/h can be considered. This value will be used for the carbon footprint assessment.

The raw materials were subjected to X-ray Fluorescence (XRF) in order to determine their chemical composition. For this purpose, ZSX PPRIMUS IV (Rigaku) equipment with a power of 4 kW was used, as well as X-ray diffraction (XRD) for all raw materials and for the samples hardened at the age of 7, 14 and 28 days in the different hardening environments. For XRD, a Bruker D8 Discover A25 instrument with Cukα (λ = 1.54050 A, 40 kV and 30 mA) was used. A speed of 0.018 2θ·s^−1^ was used from 10° to 70° (2θ). The library used to compare the crystalline peaks was the JCPDS library [60]. Thermogravimetric analysis and differential thermal analysis (TGA/DTA) were applied for all raw materials and for the hardened samples at the same ages for XRD. TGA/DTA was carried out with Setaram Setys Evolution 16/18 apparatus. Before XRF, XRD and TGA/DTA, all samples were properly powdered. The heating rate of the TGA/DTA test was 5° min^−1^, and the temperature range was approximately 20–1000 °C. At the indicated ages and for XRD and TGA/DTA, the samples were immersed in pure absolute ethanol (PanReac.AppliChem) for 48 h to “stop” the setting reactions. The same recommendations and procedures indicated by RILEM TC-238 SCM were followed, except for the immersion time (in this case 48 h) [61].

“All raw materials and hardened concretes were prepared by crushing them in advance to obtain a representative sample of each material (for XRD and TGA/DTA). The powder was quartered and all measurements were carried out in triplicate. A similar procedure was carried out in other investigations [4,62,63,64]. The compressive strength [65] was determined at 1, 3, 7, 14 and 28 days of curing in the two hardening environments presented. Dry bulk density and accessible porosity for water were determined at 28 days of age, according to UNE 83980 [66], for both environments.

### 2.5. Carbon Footprint Assessment

**Figure 5:**
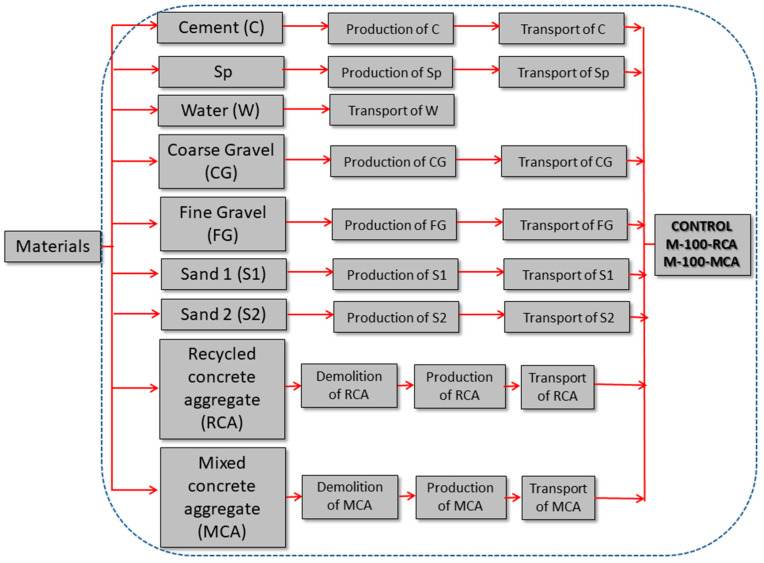
CO_2_ emission system for CONTROL, M-100-RCA and M-100-MCA mixtures.

The calculation of the carbon footprint was made for each mixture: CONTROL, M-100-RCA and M-100-MCA. As can be seen in Table 2, the main difference was the use of CG, FG, RAC or MAC. The CO_2_ emission system for the production of the different blends is presented in Figure 5.

The CO_2_ emissions: “CO_2_ emitted materials” from the materials shown in Figure 5 for 1 m^3^ of the mixture can be calculated according to Equation (8) [46].

(8) $${CO}_{2}\;emitted\;materials\;=\;\sum_{i=1}^{n}N_{i}\cdot I_{i}$$

where *n* represents the number of raw materials used (see Figure 5); N*_i_* is the weight of material used to make 1 m^3^ of concrete (kg); and I*_i_* is the CO_2_ emission of material *i* per kilogram (kg CO_2_ eq/kg).

The CO_2_ emissions produced by the curing of the samples “CO_2_ emitted by curing”, both in the CCC and CO_2_CC environment can be calculated according to Equation (9) to make 1 m^3^ of concrete [52].

(9) $${CO}_{2}\;emitted\;by\;curing\;=\;\left(E_{ele}\cdot t\right)\;{+\;(E}_{CO2-cur}\cdot t)*1\;m^{3}$$

where E_ele_ is the CO_2_ emission of electricity (kg CO_2_ eq/h) for conventional climatic chamber (CCC); E_CO2-cur_ is the CO_2_ emission of electricity for CO_2_ climatic chamber (CO_2_CC) and t hours for curing (h). If the sample was cured in CCC, only the first term of Equation (9) was taken into account. If it was cured in CO_2_CC, only the second term was considered. The emission factor for the Spanish electricity grid was considered to be 200 g CO_2_/KWh [67]. The difference between E_ele_ and E_CO2-cur_ is that the CO_2_ emission factor of E_CO2-cur_ is, albeit insignificant, slightly higher because it also considers the CO_2_ emission necessary to capture the CO_2_ used in the curing process. Normally, this capture is carried out in industries that generate CO_2_, usually using monoethanolamine (MEA) as a capture device, because of its many advantages [68,69]. Therefore, for this rough estimate, both E_ele_ and E_CO2-cur_ would be considered equal, although we want to clarify their differences. Finally, the total CO_2_ emissions would be the sum of Equations (8) and (9), as shown in Equation (10). These emission factors may differ for other investigations, as the efficiency of the equipment used may be different. However, they are usually very close to each other.

(10) $$Total\;{CO}_{2}\;emissions\;=\;{CO}_{2}\;emitted\;materials\;+\;{CO}_{2}\;emitted\;by\;curing$$

Table 3 shows the carbon emission coefficient for the different materials (I_i_) and for both curing chambers (E_ele_ and E_CO2-cur_) [41,46,47,49,70,71,72]. Emissions from the mixing process were not taken into account. The Ii factor depends, among other factors, on the fineness of the material (if it is obtained by crushing) and whether it comes from the natural or recycled aggregate.

## 3. Results and Discussion

### 3.1. Raw Materials

**Figure 6:**
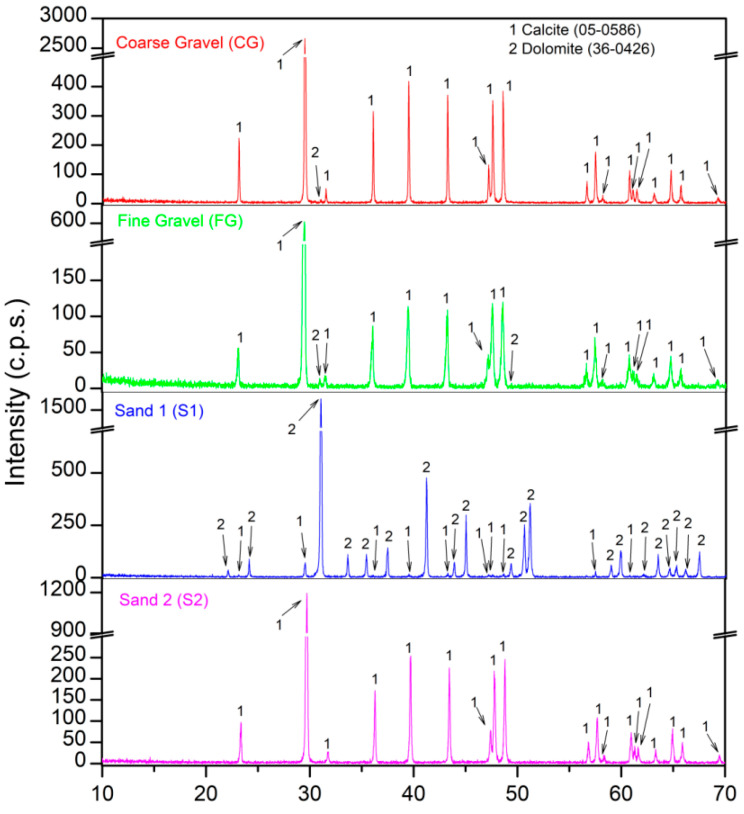
XRD patterns of natural aggregates used.

**Figure 7:**
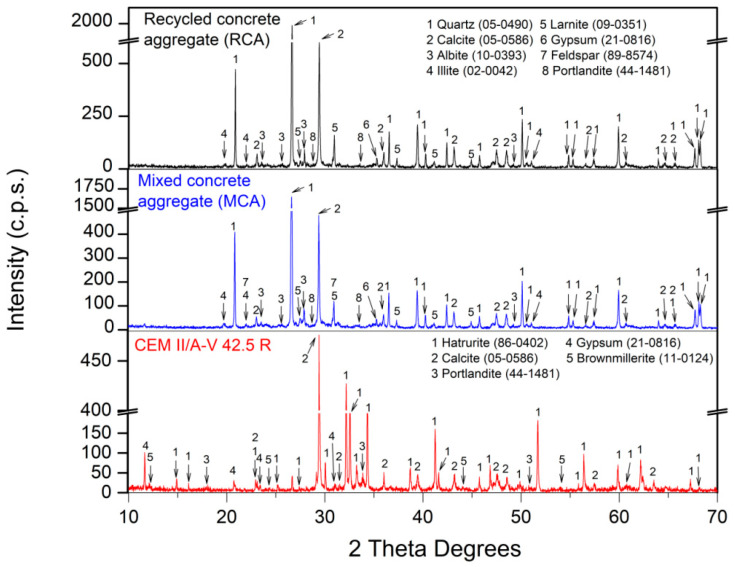
XRD patterns of recycled aggregates and cement used.

Table 4 presents the chemical composition. Figure 6 and Figure 7 show the mineralogical composition of all the raw materials used in this research. The fundamental oxide of both gravels (CG and FG) was CaO. The main phase for CG and FG was calcite (CaCO_3_) (05-0586) [60]. A very small intensity of dolomite (CaMg(CO_3_)_2_) (36-0426) [60] was also found in both gravels. For S1, the amount of CaO decreased while MgO increased, which was reflected in XRD (Figure 6), where the dolomite phase had a higher intensity than that of CG and FG. For S2, again, the major oxide was CaO and, in this case, only the calcite phase was found in XRD.

For RCA and MCA, the chemical composition was very similar. Slightly higher SiO_2_ and Al_2_O_3_ contents were found in MCA, which might be related to the content of ceramic brick pieces in MCA [73] and could improve the pozzolanic reactions in the resulting concrete [74]. The main phases for RCA and MCA were quartz (SiO2) (05-0490) [60] and calcite (CaCO_3_) (05-0586) [60]. Other minority phases were also found: albite (Na(Si_3_Al)O_8_ (10-0393) [60]; illite ((Na,K)Al_2_(Si_3_AlO_10_)(OH)_2_) (02-0042) [60]; larnite (Ca_2_SiO_4_) (09-0351) [60], gypsum (CaSO_4_·H_2_O) (21-0816) [60] and portlandite (Ca(OH)_2_) (44-1481) [60]. The portlandite phase found in RCA and MCA using X-ray diffraction is a “potential” CO_2_ sink according to Equation (3), which may be due to the fact that both RCA and MCA had been in storage for a very short time and were “fresh”. The feldspar phase (NaSiAl_3_O_8_) [60] was found in MCA and not in RCA, which may have come from the pieces of ceramic brick that present MCA [75].

**Figure 8:**
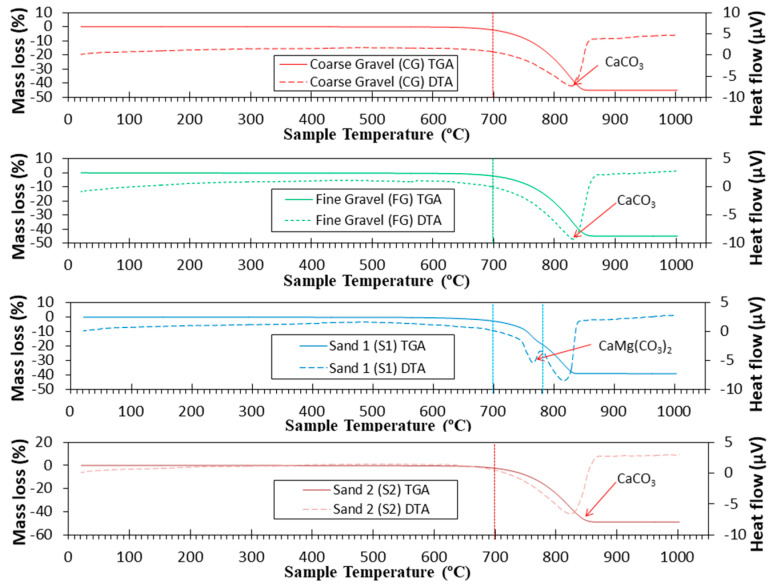
TGA (Solid lines) and DTA (dotted lines) curves for natural aggregates used.

TGA and DTA for the natural aggregates used in this study are presented in Figure 8. For CG and FG, the main mass loss started at approximately 700 °C. From this range, the decomposition of calcite started, according to Equation (11), which was the main phase found in XRD. The low dolomite intensity found in the gravels was not detected by TGA/DTA. Similar results were obtained in other research studies [12,30,76].

(11) $${CaCO}_{3}\to {CO}_{2}+CaO$$

However, for S1, a change in DTA was observed, although it did not start to lose mass noticeably until 700 °C, because dolomite was the main phase found in S1. The thermal decomposition of dolomite includes two stages [77,78]. The first stage (from 700 to 780 °C) is shown in Equation (12) and the second stage (from 780 to 1000 °C) in Equation (11).

(12) $$CaMg{(CO}_{3}{)}_{2}\to {CaCO}_{3}+MgO+{CO}_{2}$$

A very similar result was found for S2 as for FG and CG. This confirms the purity of calcite found for S2.

**Figure 9:**
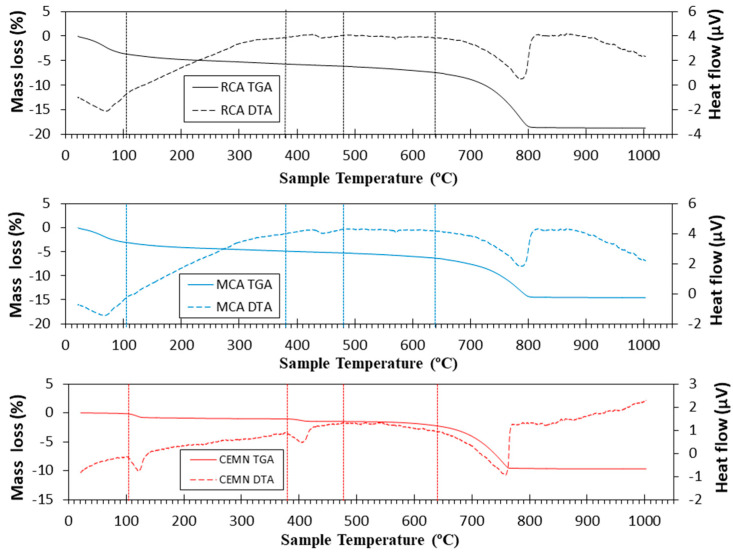
TGA (Solid lines) and DTA (dotted lines) curves of recycled aggregates and cement used.

TGA and DTA for RCA, MCA and cement used in this study are presented in Figure 9. For RCA and MCA, the TGA/DTA result was very similar: (i) up to 105 °C of the physically absorbed water was lost [30]; (ii) from 105 to 380 °C, the loss of hydrated calcium silicates and aluminates occurred (CSH and CASH, respectively) [79,80]; (iii) from 380 to 480 °C, an endothermic peak (in DTA) related to the loss of portlandite was found which was identified in XRD (Figure 7) [23,81] and (iv) from 640 to 1000 °C, decomposition of calcium carbonate occurs, according to Equation (11) [4,82]. For cement, a typical result was found, with ranges of weight loss already extensively described in other research studies [5,12,30].

### 3.2. Compressive Strength

**Figure 10:**
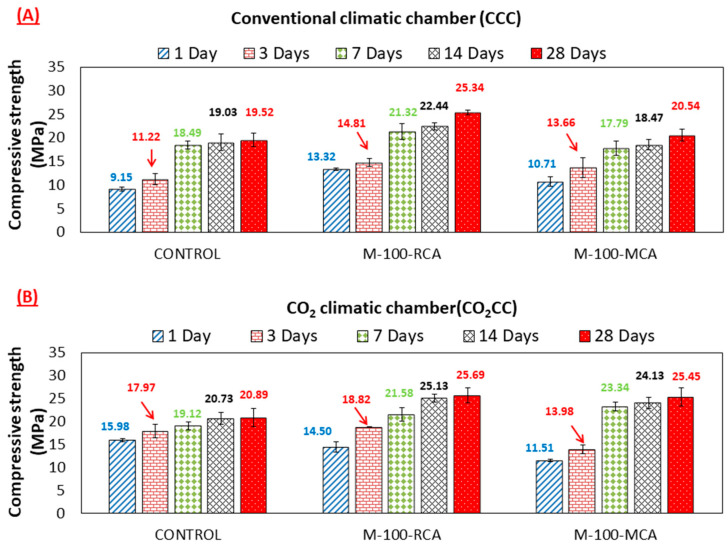
Compressive strength development of CONTROL, M-100-RCA and M-100-MCA under (**A**) CCC and (**B**) CO_2_CC at the ages of 1, 3, 7, 14 and 28 days.

Figure 10 shows the compressive strength of all the mixes studied, under both curing environments for 1, 3, 7, 14 and 28 days of age. The substitution of CG and FG for RCA using CCC (i.e., CONTROL vs. M-100-RCA) improved the compressive strength at all curing ages. Similarly, the substitution of CG and FG by MCA using CCC (i.e., CONTROL vs. M-100-MCA) also improved the compressive strength at all curing ages. These increases compared to CONTROL for the age of 28 days were 29.81 and 5.22% for M-100-RCA and M-100-MCA, respectively. While this is a very good result, it is unusual. The compressive strength of recycled aggregate was lower than conventional concrete [28,83]. There are many factors that influence the relationship between compressive strength and the use of recycled aggregates [84]: (a) recycled aggregate replacement level; (b) recycled aggregate size; (c) quality of recycled aggregate; (d) influence of the mixing procedure; (e) environmental conditions; (f) chemical admixtures and (g) additions incorporation. In this case, the use of very similar sizes between the FG and CG. Both recycled aggregates were very important factors (see Figure 3, factor (b)) [85], as well as the use of saturated aggregate before mixing (see Table 2, factor (d)) [86]. The quality of the recycled aggregate is also very important (factor (c)), as the portlandite and larnite phases found (see Figure 7 and Figure 9) can lead to improvements in compressive strength [87]. This indicates the feasibility of replacing CG and FG with RCA and MCA, which maximises the circular economy concept and minimises the use of non-renewable natural resources.

Curing with a CO_2_ climatic chamber (CO_2_CC) improved compressive strength at all ages, compared to the conventional climatic chamber (CCC). This result, which is very common, is recognised in most research [25,39,88] and is caused by a “densification” of the sample due to carbonation according to Equations (1)–(5) [21,89]. For the control mixture, 7 days of curing in CO_2_CC was similar to 28 days of curing in CCC. For the M-100-RCA mixture, 14 days of curing in CO_2_CC was similar to 28 days of curing in CCC. For the M-100-MCA mixture 7 days of curing in CO_2_CC improved by 13.53% the compressive strength obtained for 28 days of curing in CCC. These results highlight the difficulty of making comparisons between CO_2_CC and CCC curing for compressive strength purposes, as it depends on the nature of each mix. Several authors have already indicated that the mechanisms of natural and accelerated carbonation are different [23,90]. It indicates that the effect of curing in CO_2_ after 14 days was insignificant, it being possible that from this age the samples were fully carbonated (perhaps it could be indicated for 7 days, especially for the control and M-100-MCA mixture). With the mixtures studied, it is not necessary to cure for up to 28 days in CO_2_CC. Rather, 7 or 14 days are sufficient. The results obtained also indicated that CO_2_CC can be used in a non-reinforced precast plant, increasing productivity and decreasing the curing time.

### 3.3. Dry Bulk Density and Accessible Porosity for Water

**Figure 11:**
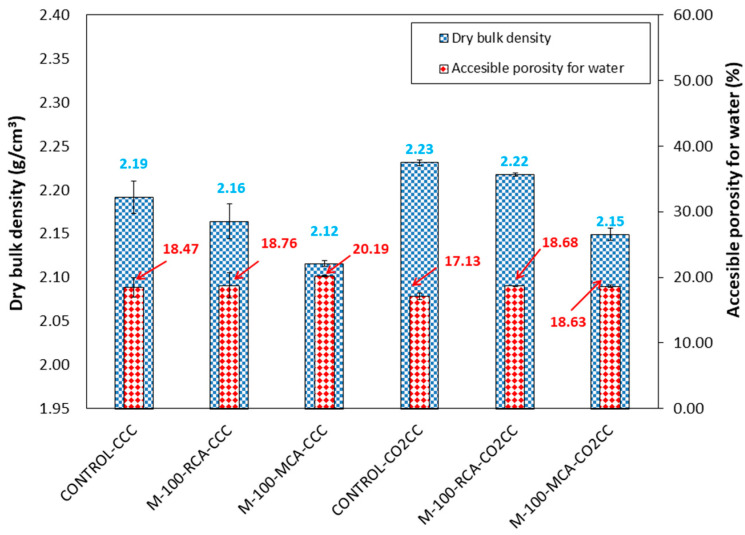
Dry bulk density and accessible porosity for water of control, M-100-RCA and M-100-MCA under CCC and CO_2_CC at 28 days.

Figure 11 shows dry bulk density and accessible porosity for water at 28 days of curing for CCC and CO_2_CC. The substitution of CG and FG for RCA and MCA decreased dry bulk density and increased accessible porosity for water for all the mixtures and under both curing environments. This was due to the lower dry bulk density and higher water absorption of RCA and MCA versus CG and FG (Table 1), mainly due to the cementitious mortar adhered to RCA and MCA surface having greater porosity than natural aggregates [91,92].

Curing in CO_2_ (CO_2_CC) increased the dry bulk density and decreased accessible porosity for water, which is in accordance with the improvement in compressive strength found (Figure 10) and was due to the pore-filling effect produced by the carbonation, in accordance with other studies [12,38,93,94].

### 3.4. X-ray Diffraction Analysis

**Figure 12:**
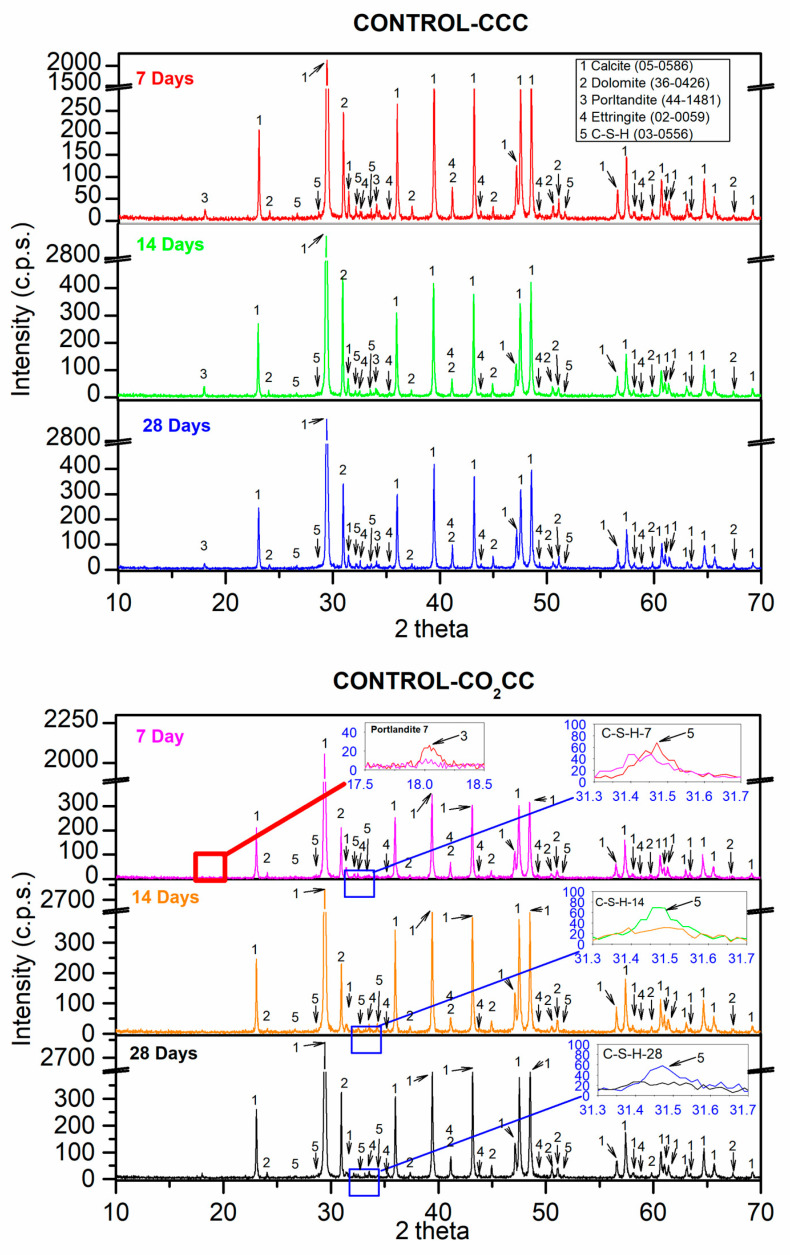
X-ray diffraction obtained for control under CCC and CO_2_CC at 7, 14 and 28 days.

Figure 12 shows the XRD results for the CONTROL mixture under the two curing environments (CCC and CO_2_CC) at ages 7, 14 and 28 days. For the mixture, the control mixture at 7 days under CCC for the main phases was calcite (CaCO_3_) (05-0586) [60] and dolomite (CaMg(CO_3_)_2_) (36-0426) [60]. These phases came from the aggregates used (CG, FG, S1 and S2). The portlandite (Ca(OH)_2_) (44-1481) [60], ettringite (Ca_6_Al_2_(SO_4_)_3_(OH)_12_·26 H_2_O) (00-0059) [60] and calcium silicate hydrate (2CaSiO_3_·3H_2_O) (03-0556), also named C-S-H [60] phases, were the main hydration reactions [95,96,97]. The same phases were found at 14 and 28 days with no significant changes. Note that at 7 days, neither the alite phase (also sometimes called hatrurite) nor the belite phase were not found. In other investigations with similar mixtures [5,12,30], they were found, especially at 1 and 3 days of curing. This was the result of the alite having reacted almost completely at 7 days, together with gypsum, which was also not found, to form ettringite, as shown in Equation (13). It is also the result of the hydration of Portland cement, according to Equations (14) and (15) [98].

(13) $$3CaO\cdot {Al}_{2}O_{3}+3{CaSO}_{4}+32H_{2}O\to 3CaO\cdot {Al}_{2}O_{3}\cdot 3{CaSO}_{4}\cdot 32H_{2}O$$

(14) $$2\left(3CaO\cdot {SiO}_{2}\right)+6H_{2}O\to 3CaO\cdot 2{SiO}_{2}\cdot 3H_{2}O+3Ca{\left(OH\right)}_{2}$$

(15) $$2\left(2CaO\cdot {SiO}_{2}\right)+4H_{2}O\to 3CaO\cdot 2{SiO}_{2}\cdot 3H_{2}O+Ca{\left(OH\right)}_{2}$$

Following the above, this indicates that applying accelerated carbonation after 7 days of curing to the control mix will be less “efficient”, as Equations (1)–(5) would be reduced to Equations (3)–(5). This was in accordance with the slight improvements found after 7 days in the compressive strength for the control mix in Figure 10, under CO_2_CC.

Basically, carbonation at ambient pressure primarily includes three steps: (1) diffusion of CO_2_ producing CO_3_^2−^; (2) dissolution of calcium-based phases, generating Ca^2+^ and (3) nucleation and precipitation of CaCO_3_ according to Equations (1)–(5) [99,100]. Applied to the phases found, carbonation applied with CO_2_CC should “consume” phases such as portlandite and C-S-H Equations (3)–(5). In fact, at 7 days, under CO_2_CC it can be observed in the inset labelled “Portlandite 7” that the portlandite phase has practically disappeared (red line vs pink line). Additionally, a slight decrease in the C-S-H phase was observed when CO_2_CC was used. This is indicated in the inset labelled “C-S-H-7” (red line vs pink line). The rest of the phases found were the same as those found under CCC, although perhaps with a little more intensity in the calcite phase, which is in accordance with Equations (3)–(5). At 14 and 28 days, under CO_2_CC, the portlandite phase was logically still absent. As for the C-S-H phase, the decrease produced by the contact of this phase with CO_2_ is fulfilled, which can be observed in the inset “C-S-H 14” and “C-S-H 28” respectively for the age of 14 and 28 days.

**Figure 13:**
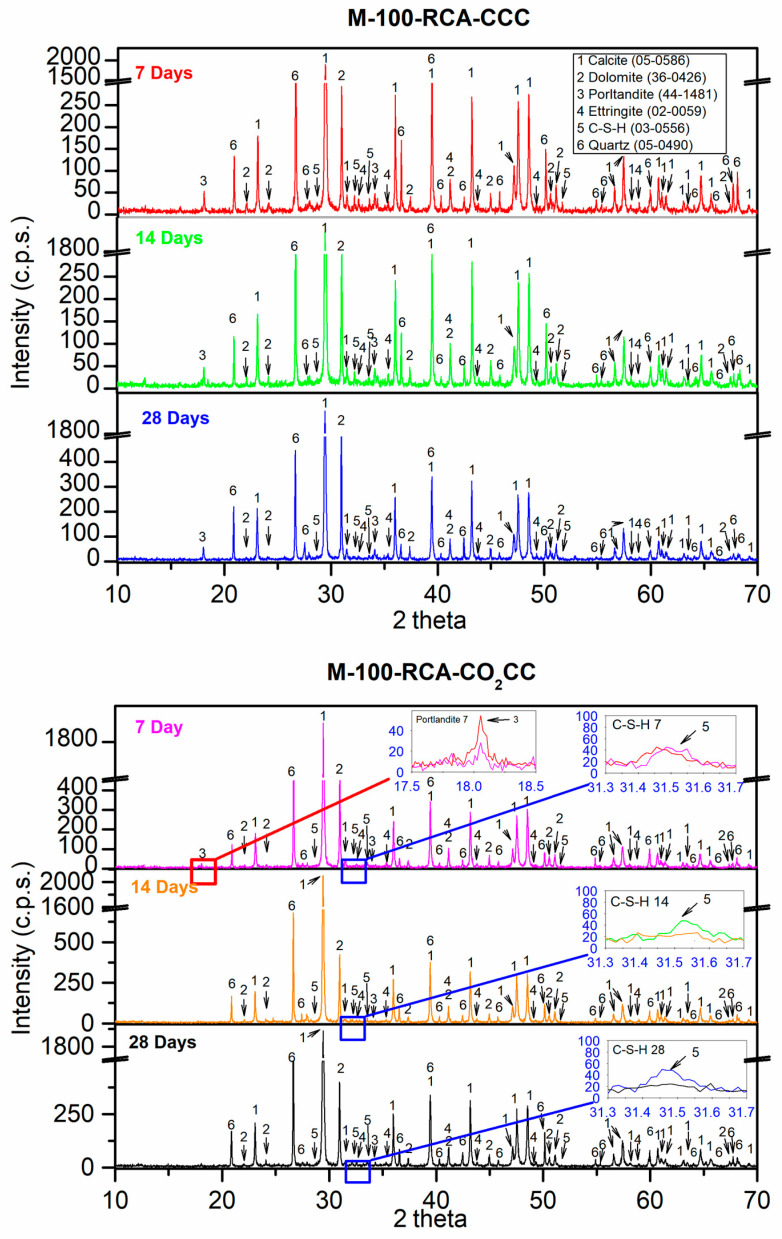
X-ray diffraction obtained for M-100-RCA under CCC and CO_2_CC at 7, 14 and 28 days.

Figure 13 shows the XRD results for the M-100-RCA mixture under the two curing environments (CCC and CO_2_CC) at 7, 14 and 28 days. For the mixture M-100-RCA at 7 days under CCC, the main phases were calcite (CaCO_3_) (05-0586) [60] and dolomite (CaMg(CO_3_)_2_) (36-0426) [60]. These phases came from S1 and S2 (Figure 6). The quartz (SiO_2_) (05-0490) [60], coming from the use of RCA, also appeared as a main phase (Figure 7). As with the control mixture, the phases of portlandite (Ca(OH)_2_) (44-1481) [60], ettringite (Ca_6_Al_2_(SO_4_)_3_(OH)_12_·26 H_2_O) (00-0059) [60] and calcium silicate hydrate (2CaSiO_3_·3H_2_O) (03-0556) [60] were found. No changes were found at 14 and 28 days cured in CCC. The absence of alite and belite that was found in other research of similar blends [5,12,30] is indicative that “it makes no sense” to apply carbonation at 7 days of curing.

Under CO_2_CC, at 7 days, it can be observed in the inset labelled “Portlandite 7” that this phase still exists, although with little intensity. For the control mixture, this phase did not exist. This may be due to the portlandite already present in the RCA itself (Figure 7). Therefore, this may be indicative that the use of RCA with the portlandite phase is a “CO_2_ sink” according to Equation (3). Again, a small decrease in the C-S-H phase was also observed, indicated by the inset labelled “C-S-H 7” (red line vs. pink line). At 14 days, the disappearance of the portlandite phase was observed, which is in accordance with the fact that after 14 days the compressive strength remained approximately constant, and there was no significant increase (Figure 9). Logically, the same is true at 28 days of curing. The insets labelled “C-S-H 14” and “C-S-H 28” show the same as those obtained for the control mix.

**Figure 14:**
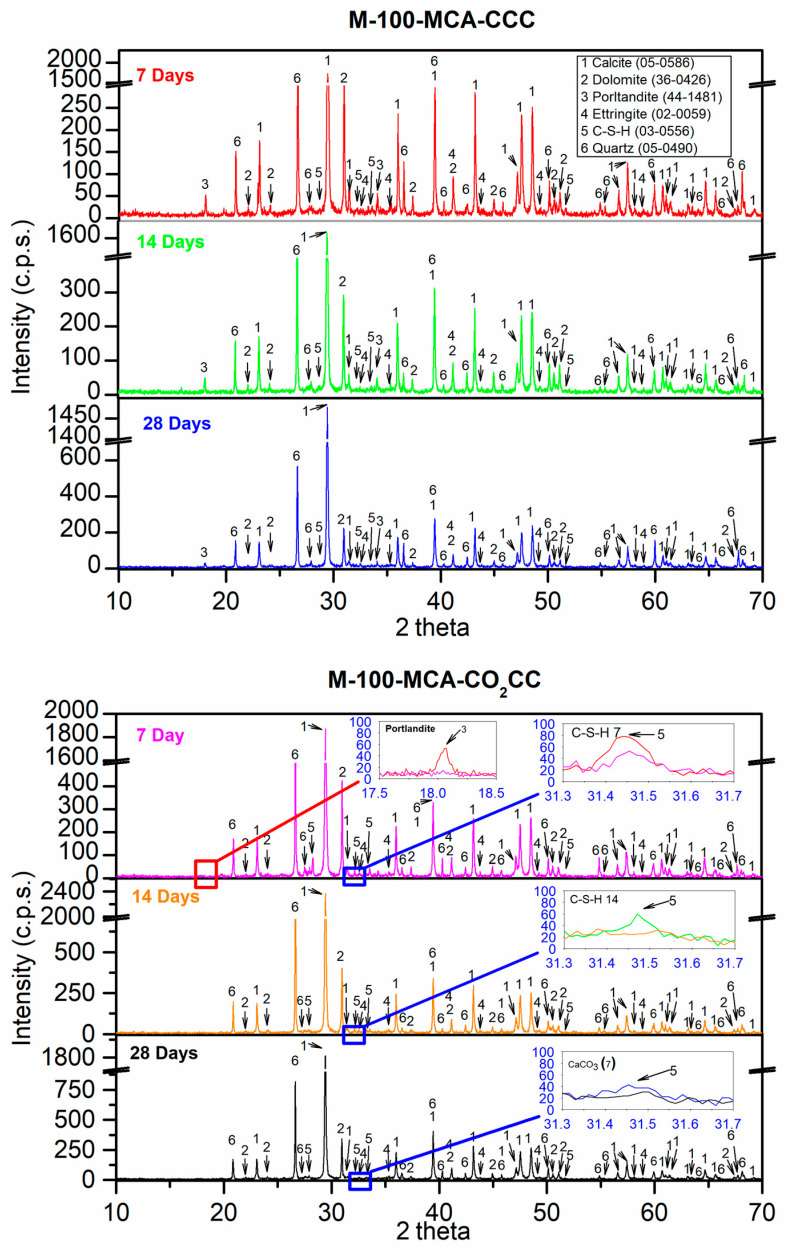
X-ray diffraction obtained for M-100-MCA under CCC and CO_2_CC at 7, 14 and 28 days.

Figure 14 shows the XRD results for the M-100-RCA mixture under the two curing environments (CCC and CO_2_CC) at 7, 14 and 28 days. For the mixture M-100-MCA at 7 days under CCC, the main phases were calcite (CaCO_3_) (05-0586) [60], dolomite (CaMg(CO_3_)_2_) (36-0426) [60] and quartz (SiO_2_) (05-0490) [60]. Other minority phases were also found: portlandite (Ca(OH)_2_) (44-1481) [60], ettringite (Ca_6_Al_2_(SO_4_)_3_(OH)_12_·26 H_2_O) (00-0059) [60] and calcium silicate hydrate (2CaSiO_3_·3H_2_O) (03-0556) [60]. These phases were also found in the control and M-100-MCA mixtures. Again, belite and alite phases were not found, which is again indicative that it is not necessary to carbonate this type of sample at over 7 days of curing.

Under CO_2_CC, at 7 days it can be observed in the inset labelled “Portlandite 7” that the portlandite phase has practically disappeared (red line vs pink line). Also, a slight decrease in the C-S-H phase was observed when CO_2_CC was used. This is indicated in the inset labelled “C-S-H-7” (red line vs pink line). At 14 and 28 days, under CO_2_CC, the portlandite phase is logically still absent. As for the C-S-H phase, the decrease produced by the contact of this phase with CO_2_ is fulfilled, which can be observed in the inset “C-S-H 14” and “C-S-H 28” respectively at 14 and 28 days.

### 3.5. Thermogravimetric Analysis and Differential Thermal Analysis

**Figure 15:**
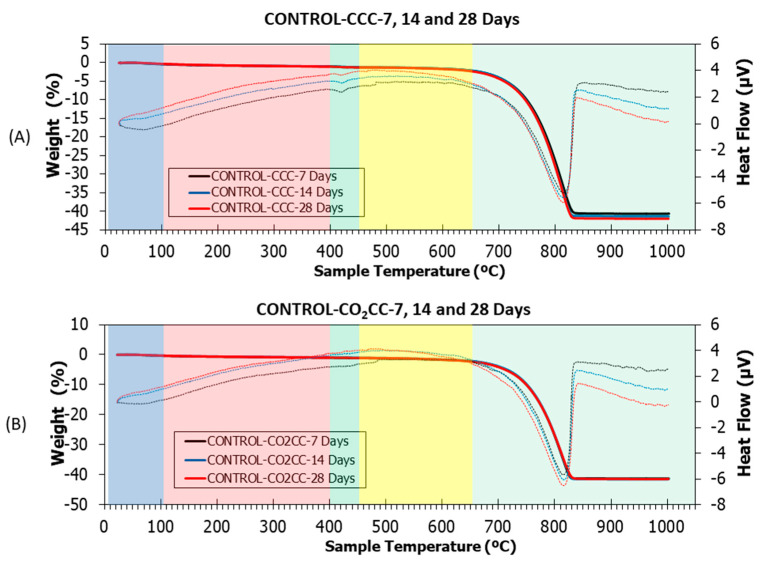
TGA (solid lines) and DTA (dotted lines) of control under (**A**) CCC and (**B**) CO_2_CC at 7, 14 and 28 days.

**Figure 16:**
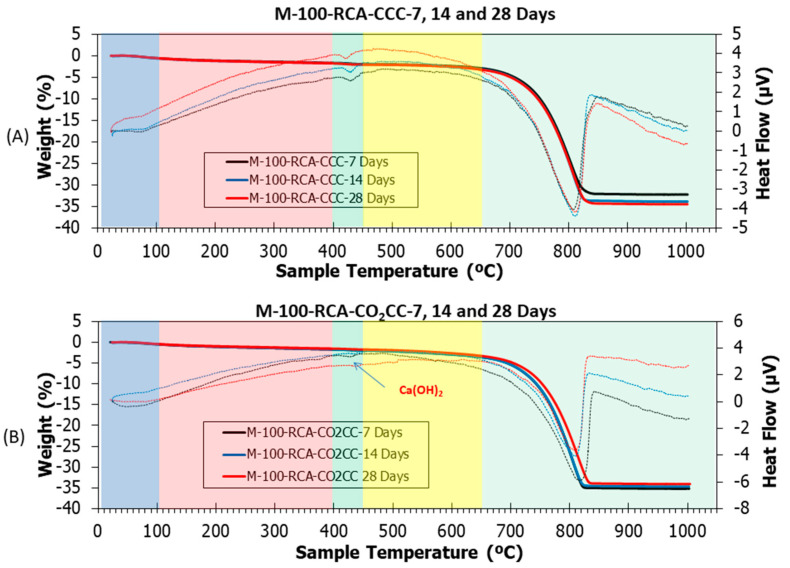
TGA (solid lines) and DTA (dotted lines) of M-100-RCA under (**A**) CCC and (**B**) CO_2_CC at 7, 14 and 28 days.

**Figure 17:**
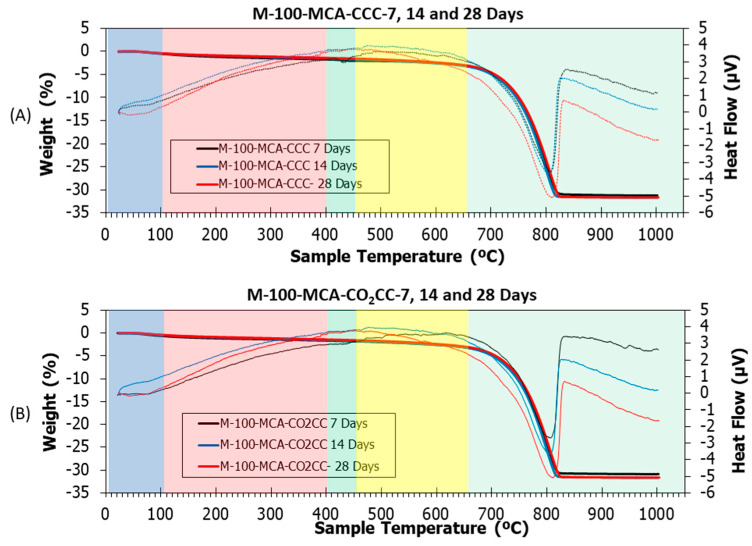
TGA (solid lines) and DTA (dotted lines) of M-100-MCA under (**A**) CCC and (**B**) CO_2_CC at 7, 14 and 28 days.

Figure 15, Figure 16 and Figure 17 show thermogravimetric analysis (TGA) and differential thermal analysis (DTA) of all samples studied under CCC and CO_2_CC. Table 5 shows the weight losses for the different stretches. This analysis can determine the CO_2_ absorption produced through CO_2_ curing [101,102] by just comparing the amount of calcium carbonate in the same mixture before and after curing in CO_2_, as indicated in Equation (16). This can be done because the main product of carbonation is CaCO_3_, as indicated in Equations (1)–(5).

(16) $${CO}_{2}\;sequestrated\;\left(wt.\%\right)\;=\;{CaCO}_{3}\;in\;{CO}_{2}CC\;-\;{CaCO}_{3}\;in\;CCC$$

Several common stretches were found in all the samples studied [5,12,30]: (i) From room temperature to 105 °C, ambient humidity was lost. It was observed that this loss was higher in the M-100 RCA and M-100-MCA mix than in the control mix, both for CCC and CO_2_CC, due to the higher water absorption of both RCA and MCA compared to CG and FG, as indicated in Table 1; (ii) From 105 to 400 °C, loss of ettringite and C-S-H detected by XRD occurred [103,104,105]. A slight decrease in weight loss was observed when comparing CCC versus CO_2_CC, which was already detected by XRD (see insets named “CSH 7”, “CSH 14” and “CSH 28”) for all samples; (iii) From 400 to 460 °C, decomposition of portlandite, if present, occurred. Its existence was identified with an endothermic peak in DTA. Under CCC, portlandite existed in all samples, as demonstrated by XRD. However, under CO_2_CC, it only appears in sample M-100-RCA at 7 days, which is in accordance with the XRD findings. The weight loss (Table 5) was lower in CO_2_CC than in CCC, which is in accordance with the above; (iv) From 460 to 650 °C, the initial carbonates formed in the hardening process were lost [106] and from 650 to 1000 °C, the calcium carbonate was lost [82], which is in accordance with the remarkable weight loss found. In this study, we will consider the last two sections together.

In order to observe the effect of CO_2_ curing, a comparison was made between CCC and CO_2_CC for the same samples and curing ages (Table 5). Generically, it was observed that the capture capacity increases under the CO_2_CC curing environment, which is indicative that CO_2_ curing of these materials not only improves compressive strength, but also absorbs CO_2_.

The increase for the control mix was 2.9, 3.6 and 3.6 kg/CO_2_ t sample under CO_2_CC. Two aspects are derived from the above: Firstly, it can be seen that using CO_2_CC longer than 14 days does not make sense from the point of view of increasing CO_2_ absorption. Secondly, it may appear as a low CO_2_ uptake. However, considering that the average CO_2_ level in the atmosphere is 400 ppm and, considering that the density of CO_2_ is 1.8 mg/cm^3^ under normal conditions, the amount of CO_2_ in 1 m^3^ of air is only 721.6 mg [107]. To reach the CO_2_ level of the pre-industrial era (280 ppm), only absorbing 120 ppm would be sufficient, i.e., 216.48 mg per m^3^ of air. With 1 tonne of the control mixture, 13,604 m^3^ of air could be decarbonised after only 7 days of curing.

For the M-100-RCA mixture, again it can be seen that it does not make sense to cure in CO_2_CC for more than 14 days. In this case, the CO_2_ absorption is higher than for the control mix, and this is due to the portlandite phase found in the RCA aggregate (Figure 7). Therefore, with only 7 days of curing, the 1 tonne of M-100-RCA mix could decarbonise under a CO_2_CC environment of about 36,077 m^3^ of air. It also does not make sense from a CO_2_ absorption point of view to cure the M-100-MCA mix for more than 14 days. In this case, 7 days of curing of 1 tonne of M-100-MCA mix would decarbonise 24,685 m^3^ of air. Considering that a conventional cobblestone has dimensions of 20 × 10 × 6, and using the dry densities obtained in Figure 11 as an estimate, the following was obtained: 1 single paving stone could decarbonise 3.61, 9.59 and 6.56 m^3^ of air at pre-industrial levels with the control, M-100-RCA and M-100-MCA mixtures, respectively.

Furthermore, the increase in CO_2_ sequestered per m^3^ has been calculated using the dry densities obtained in Figure 11. This will be used to calculate the carbon emission evaluation.

Based on the authors’ knowledge, no studies have been found that study the CO_2_ capture capacity of concrete mixes such as the one presented. They were found for very porous mortars, using similar levels of carbonation, but only using 7 days of curing [12], using carbonated water as curing and/or mixing water [5,20]. In other research, although CO_2_ is calculated, it is only estimated qualitatively [108]. This research fills this information gap.

### 3.6. Carbon Emission Evaluation

According to Table 2, Table 3 and Table 5, as well as Equations (8) and (9), the carbon emissions of each of the mixtures with different materials and curing were assessed and are presented in Table 6. Table 7 shows the results obtained for the incremental capture per m^3^ calculated through TGA/DTA (Table 5) with the respective carbon emissions calculated in Table 6.

CO_2_ emitted materials amounted to 223.18, 216.68 and 216.50 kg CO_2_ eq/m^3^ respectively for the control, M-100-RCA and M-100-MCA mixtures, respectively. This result is slightly below the range shown by W. Xing et al. [49] (278.35–524.44 kg CO_2_ eq/m^3^) in a review where they studied the environmental impact of 57 concrete products. These differences are caused by the effect of substituting fine or coarse aggregate [109,110], the recycling process [111,112] and the source and quality of primary material [113], among others. Furthermore, this result showed the feasibility of replacing CG and FG (natural aggregates) with RCA and MCA (recycled aggregates from construction and demolition waste).

However, it was observed that CO_2_ emissions for curing for 14 and 28 days were quite high (at least with the equipment used at the laboratory scale). In fact, for the control mix, CO_2_ emissions for curing at 14 and 28 days (10.08 and 20.16 kg CO_2_ eq/m^3^ shown in Table 6), are higher than the increase in CO_2_ sequestrated (8.23 and 8.16 kg CO_2_ sequestrated by m^3^ shown in Table 5). This again shows that, for the control mix it, does not make sense to cure more than 7 days in CO_2_CC. Using the same comparison, for the M-100-RCA and M-100-MCA mixtures, the CO_2_ emissions for curing would allow curing in CO_2_CC for up to 14 days. It is therefore not feasible to cure for 28 days in CO_2_CC for any of the mixtures studied.

Therefore, a CO_2_CC cure of up to 7 days (or even less) was sufficient to use CO_2_ curing as a tool to increase productivity, improve compressive strength and decrease CO_2_ emission in an unreinforced precast plant. It is not recommended to use more than 7 days in CO_2_ curing, as compressive strengths were maintained and only CO_2_ emissions were increased. No studies were found that relate the CO_2_ sequestered by similar mixtures and that also make an assessment of CO_2_ emissions, relating both results.

The mixture with the lowest total CO_2_ emissions was “M-100-RCA-CO_2_CC- 7 Days”. According to the strengths obtained in Figure 10, it could be classified as C16/20 “ordinary concrete” only at 7 days [57]. Therefore, “M-100-RCA-CO_2_CC- 7 Days” presents a very promising result to be tested on a real scale, such as paving stones, kerbs or any non-structural precast.

## 4. Conclusions

CO_2_ curing in vibro-compacted precast concrete with recycled/mixed concrete aggregates (RCA or MCA) is a promising technology. The CO_2_ curing enabled carbon sequestration, improved the compressive strength, increased the dry bulk density and decreased the accessible porosity for water. The findings are detailed in the following points:

- The portlandite phase found in RCA and MCA by XRD is a “potential” CO_2_ sink;
- The method of replacing natural aggregate with RCA and MCA should be carried out with very similar particle sizes. This even improves the compressive strengths obtained;
- Curing in CO_2_ improved the compressive strength in all samples (CONTROL, M-100-RCA and M-100-MCA). It does not make sense to apply CO_2_ curing longer than 7 days on the mixes with natural aggregate and MCA, as the strengths remained constant. A CO_2_ curing of 14 days can be applied to the RCA mixes;
- XRD and TGA/DTA showed that it does not make sense to apply CO_2_ curing beyond 7 days, since from that age all the blends were practically carbonated (except the blend with RCA, which did not carbonate until 14 days);
- The mixtures of 1 tonne of control, M-100-RCA and M-100-MCA using CO_2_ curing could be decarbonised after only 7 days of curing 13,604, 36,077 and 24,635 m^3^ of air, respectively;
- According to the carbon emission evaluation and the TGA/DTA results, curing longer than 7 days in CO_2_ for the reference mix (CONTROL) had higher CO_2_ emissions than the sequestered CO_2_. The mix with RCA and MCA would allow up to 14 days, but according to the compressive strength obtained; XRD and TGA/DTA results, only up to 7 days is recommended;
- The total CO_2_ emissions by mixture using CO_2_ curing at 7 days were 221.26, 204.38 and 210.05 kg CO_2_ eq/m^3^ for CONTROL, M-100-RCA and M-100-MCA, respectively. This was calculated with the carbon footprint assessment and the CO_2_ sequestrated obtained with TGA/DTA.

In conclusion, the findings of this study provide a valuable contribution to carbon emission evaluation of CO_2_ curing in vibro-compacted precast concrete with recycled/mixed concrete aggregates (RCA or MCA). The new approach facilitates carbon capture and use and guarantees enhanced compressive strength of the concrete samples.

## Figures and Tables

**Table 1:** Basic physical parameters of the aggregates used.

Type of Aggregates	Skeletal Density γ (g/cm^3^)	Water Absorption (%)
Coarse gravel (CG)	2.47	3.13
Fine gravel (FG)	2.43	2.64
Sand 1 (S1)	2.65	2.40
Sand 2 (S2)	2.62	1.78
Recycled concrete aggregate (RCA)	2.21	7.42
Mixed concrete aggregate (MCA)	2.17	9.02

**Table 2:** Mixture proportion of concrete mixtures.

Notation	Mixture Proportions (kg/m^3^)										
	Effective Water	Additional Water/Saturati-on water	Cem	Coarse Gravel (CG)	Fine Gravel (FG)	Sand 1 (S1)	Sand 2 (S2)	Recycled Aggregate Concrete (RAC)	Mixed Aggregate Concrete (MAC)	Sp	W/C
CONTROL	84	50	210	200	700	200	1200	-	-	0.5	0.4
M-100-RCA	84	86.70	210	-	-	200	1200	815.60	-	0.5	0.4
M-100-MCA	84	98.22	210	-	-	200	1200	-	798.95	0.5	0.4

**Table 3:** Carbon emission coefficient for materials and curing used.

Materials	Factor Ii	Unit	References
Cement	1.002	Kg CO_2_ eq/kg	[47,49,70]
Superplasticizer	1.150	Kg CO_2_ eq/kg	[49]
Water	3.47 × 10^−4^	Kg CO_2_ eq/kg	[70]
Coarse gravel	4.10 × 10^−3^	Kg CO_2_ eq/kg	[47]
Fine gravel	9.87 × 10^−3^	Kg CO_2_ eq/kg	[49]
Sand 1	2.79 × 10^−3^	Kg CO_2_ eq/kg	[71]
Sand 2	3.21 × 10^−3^	Kg CO_2_ eq/kg	[49]
Recycled concrete aggregate	1.50 × 10^−3^	Kg CO_2_ eq/kg	[46,49]
Mixed concrete aggregate	1.30 × 10^−3^	Kg CO_2_ eq/kg	[46,49]
Curing	Factor E_ele_/E_CO_2_-cur_	Unit	
Conventional Chamber (0.15 kW/h)	0.03	kg CO_2_ eq/h curing	[52,67,68,69]
CO_2_ Chamber (0.15 kW/h)	0.03	kg CO_2_ eq/h curing	[52,67,68,69]

**Table 4:** Chemical components of raw materials.

Components (Mass% as Oxide)	Coarse Gravel (CG)	Fine Gravel (FG)	Sand 1 (S1)	Sand 2 (S2)	Recycled Concrete Aggregate (RCA)	Mixed Concrete Aggregate (MCA)	Cement
Na_2_O	-	-	-	-	0.82	0.82	0.24
MgO	0.88	0.96	37.98	0.78	2.77	3.11	1.33
Al_2_O_3_	0.20	0.73	0.06	0.96	7.78	10.49	3.73
SiO_2_	0.39	2.12	0.91	2.16	51.40	52.08	15.58
P_2_O_5_	-	-	-	-	0.11	0.12	0.09
SO_3_	0.07	0.10	0.11	0.11	1.14	1.35	4.79
Cl_2_O_3_	-	0.05	0.21	-	0.06	0.10	0.18
K_2_O	0.03	0.09	0.05	0.18	1.80	2.38	1.21
CaO	98.31	95.75	60.44	90.32	30.62	25.15	70.03
TiO_2_	-	-	-	-	0.43	0.55	0.23
MnO_2_	-	-	-	-	0.09	0.08	0.06
Fe_2_O_3_	0.10	0.18	0.23	5.50	2.76	3.59	2.44
CuO	-	-	-	-	-	-	-
ZnO	-	-	-	-	-	-	0.02
SrO	0.03	-	-	-	0.03	0.04	0.08
Rb_2_O	-	-	-	-	-	0.01	-
Cr_2_O_3_	-	-	-	-	0.21	0.15	-

**Table 5:** Different weight losses for all the mixtures studied and increase in CO_2_ sequestrated using CO_2_CC.

Mixes	Δ Mass (%)					Δ Mass (450–1000 °C)	CO_2_ Sequestrated (wt.%) According to Equation (16)	Increase in CO_2_ Sequestrated (g/t)	Increase in CO_2_ Sequestrated (g/m^3^)
	RT-105 °C	105–400 °C	400–450 °C	450–650 °C	650–1000 °C				
CONTROL-CCC- 7 Days	−0.465	−0.585	−0.230	−0.879	−38.615	−39.494			
CONTROL-CCC- 14 Days	−0.414	−0.609	−0.186	−0.927	−39.351	−40.278			
CONTROL-CCC- 28 Days	−0.366	−0.734	−0.186	−1.028	−39.775	−40.804			
CONTROL-CO_2_CC- 7 Days	−0.412	−0.526	−0.155	−1.058	−38.730	−39.789	0.2945	2945.21	6567.83
CONTROL-CO_2_CC- 14 Days	−0.320	−0.588	−0.096	−1.087	−39.559	−40.647	0.3693	3693.83	8237.24
CONTROL-CO_2_CC- 28 Days	−0.326	−0.647	−0.009	−1.175	−39.995	−41.170	0.3663	3663.32	8169.12
M-100-RCA-CCC- 7 Days	−0.617	−1.086	−0.261	−0.955	−29.467	−30.422			
M-100-RCA-CCC- 14 Days	−0.698	−1.244	−0.282	−1.083	−30.912	−31.996			
M-100-RCA-CCC- 28 Days	−0.639	−1.164	−0.260	−1.144	−31.416	−32.561			
M-100-RCA-CO_2_CC- 7 Days	−0.584	−1.081	−0.205	−1.665	−29.538	−31.203	0.7810	7810.94	17,340.28
M-100-RCA-CO_2_CC- 14 Days	−0.526	−1.097	−0.198	−1.644	−31.197	−32.842	0.8457	8457.52	18,775.57
M-100-RCA-CO_2_CC- 28 Days	−0.516	−1.051	−0.175	−1.593	−31.817	−33.410	0.8492	8492.99	18,854.44
M-100-MCA-CCC- 7 Days	−0.563	−1.186	−0.289	−1.007	−28.325	−29.332			
M-100-MCA-CCC- 14 Days	−0.444	−1.116	−0.229	−1.388	−28.663	−30.051			
M-100-MCA-CCC- 28 Days	−0.611	−1.022	−0.188	−1.120	−28.306	−29.427			
M-100-MCA-CO_2_CC- 7 Days	−0.669	−0.987	−0.209	−1.317	−28.550	−29.867	0.5344	5344.98	11,491.71
M-100-MCA-CO_2_CC- 14 Days	−0.490	−0.953	−0.160	−1.530	−29.296	−30.826	0.7705	7702.25	16,652.22
M-100-MCA-CO_2_CC- 28 Days	−0.467	−0.945	−0.148	−1.440	−28.757	−30.197	0.7745	7745.27	16,560.97

**Table 6:** CO_2_ emissions for materials and Curing.

Materials	CO_2_ Emission Control (kg CO_2_ eq/m^3^)	CO_2_ Emission M-100-RCA (kg CO_2_ eq/m^3^)	CO_2_ Emission M-100-MCA (kg CO_2_ eq/m^3^)
Cement	210.420	210.420	210.42
Superplasticizer	0.575	0.575	0.575
Water	0.046	0.059	0.063
Coarse gravel	0.820	-	-
Fine gravel	6.909	-	-
Sand 1	0.558	0.558	0.558
Sand 2	3.852	3.852	3.852
Recycled concrete aggregate	-	1.223	-
Mixed concrete aggregate	-	-	1.038
CO_2_ emitted materials	223.180	216.687	216.506
Curing	7 Days (kg CO_2_ eq/m^3^)	14 Days (kg CO_2_ eq/m^3^)	28 Days (kg CO_2_ eq/m^3^)
Conventional Chamber (0.15 kW/h)	5.04	10.08	20.16
CO_2_ Chamber (0.15 kW/h)	5.04	10.08	20.16

**Table 7:** Summary of total CO_2_ emissions by mixtures and CO_2_ sequestration.

	Total CO_2_ Emissions (kg CO_2_ eq/m^3^)	Total CO_2_ Emissions—CO_2_ Sequestrated (kg CO_2_ eq/m^3^)
CONTROL-CCC- 7 Days	228.22	228.22
CONTROL-CCC- 14 Days	233.26	233.26
CONTROL-CCC- 28 Days	243.34	243.34
CONTROL-CO_2_CC- 7 Days	228.22	221.66
CONTROL- CO_2_CC- 14 Days	233.26	225.02
CONTROL- CO_2_CC- 28 Days	243.34	235.17
M-100-RCA-CCC- 7 Days	221.72	221.72
M-100-RCA-CCC- 14 Days	226.76	226.76
M-100-RCA-CCC- 28 Days	236.84	236.84
M-100-RCA-CO_2_CC- 7 Days	221.72	204.38
M-100-RCA-CO_2_CC- 14 Days	226.76	207.99
M-100-RCA-CO_2_CC- 28 Days	236.84	217.99
M-100-MCA-CCC- 7 Days	221.54	221.54
M-100-MCA-CCC- 14 Days	226.58	226.58
M-100-MCA-CCC- 28 Days	236.66	236.66
M-100-MCA-CO_2_CC- 7 Days	221.54	210.05
M-100-MCA-CO_2_CC- 14 Days	226.58	209.93
M-100-MCA-CO_2_CC- 28 Days	236.66	220.10

## Data Availability

Not applicable.
